# Research on Ship-Borne Wave Observation Experiment Based on Stereoscopic Vision

**DOI:** 10.3390/s26030993

**Published:** 2026-02-03

**Authors:** Aolong Zhu, Kefeng Mao, Li Ding, Yan Li

**Affiliations:** 1 College of Advanced Interdisciplinary Studies, National University of Defense Technology, Nanjing 211101, China; z15634137358@163.com (A.Z.); maokefeng@nudt.edu.cn (K.M.); 2 School of Marine Technology and Geomatics, Jiangsu Ocean University, Lianyungang 222005, China; 3 School of Mathematics and Statistics, Nanjing University of Science and Technology, Nanjing 210094, China; 4 Jiangsu Institute of Marine Resources Development, 59 Cangwu Road, Haizhou, Lianyungang 222005, China

**Keywords:** stereoscopic imaging, ship-borne wave measurement, Wave Acquisition Stereo System

## Abstract

Currently, most wave observation equipment is used for fixed-point measurements, and there is a relative scarcity of ship-borne real-time wave measurement devices, which limits comprehensive and three-dimensional monitoring of wave characteristics. This paper introduces the Wave Acquisition Stereo System (WASS) and describes the design and construction of a ship-borne stereoscopic vision experimental apparatus. Sea trials were conducted to evaluate the system’s ship-borne wave-measurement performance and to quantify the effect of deployment parameters on accuracy. The results indicate that the device reliably retrieves wave parameters; compared with concurrent buoy observations, the error in significant wave height did not exceed 0.14 m. Research confirms that deployment parameters have a significant impact on measurement outcomes: sampling frequency directly affects the accuracy of wave-parameter estimation; a higher sampling rate (10 Hz) improves the reliability of the calculated results. The baseline-to-height ratio has an optimal range (0.1–0.3), and values outside this interval reduce measurement accuracy. Under a fixed geometric configuration, the observation field exhibits a band-shaped low-error zone aligned with the baseline direction.

## 1. Introduction

Ocean wave observations are essential for marine disaster mitigation, maritime safety, and climate research [1]. Common approaches include remote sensing inversion, instrumental measurement, and manual observation [2]. However, traditional wave measurement techniques continue to exhibit certain limitations. For example, X-band radar offers wide-area coverage and supports directional wave-spectrum retrievals; however, the systems are costly, and the measurements are sensitive to wind-field intensity, wave breaking, and related factors [3,4]. Visual observations of sea waves require a high level of professional expertise from observers and are significantly influenced by subjective factors. Buoys provide long-term, continuous time-series data but are limited by their singular point of measurement; moreover, their deployment and retrieval processes are relatively complex [5]. Rapidly evolving marine environments (especially coastal–open-ocean transition zones and extreme storms) impose stringent requirements on observation technologies: they must maintain accuracy while delivering a wide field of view, high spatiotemporal resolution, and adaptability across platforms and challenging sea states. To meet these requirements, non-contact, scalable wave-measurement methods with three-dimensional reconstruction capability are needed to overcome the limitations of conventional techniques in handling complex sea states, supporting multiple platforms, and capturing fine-scale wave processes.

In response to these observation needs, stereoscopic vision has evolved into a crucial technology for acquiring spatiotemporal elevation fields of the ocean surface [6]. As an advanced image processing and analysis technique, it enables the retrieval of three-dimensional wave information through binocular imaging, with advantages such as real-time capability and non-contact measurement [7]. Simultaneously, as an emerging instrument measurement method [8], the application of stereoscopic vision technology in ship-based or shore-based environments allows for high-precision, all-around monitoring of ocean waves [9].

The history of using stereoscopic vision technology to observe sea waves dates back approximately a century. Schumacher pioneered this approach by installing a stereoscopic photographic system on an ocean-going vessel in 1939 [10]. Subsequently, research in the field of stereoscopic photography of sea waves was advanced by Cote and colleagues in 1954 [11]. In 1989, Banner et al. utilized stereophotogrammetry to determine the shape of the wave number spectrum [12]. Later, Zhang Suoping captured information on wave period, wavelength, wave speed, and wave direction through video images recorded by cameras [13]. Since Schumacher’s pioneering work and its subsequent applications, the integration of image analysis technology with available computational resources has significantly evolved. In recent years, stereoscopic vision analysis has emerged as a mature technique for the remote measurement of sea waves. Benetazzo and others have combined binocular vision technology with image analysis techniques to extract sea surface heights of small-scale waves from CCD images. Furthermore, surface wave information from sea waves was obtained from a set of synchronized and continuous video images captured by stereo cameras, demonstrating a technique known as the Wave Acquisition Stereo System (WASS) [6]. The technique was applied to the observation platform on the Venice coastline [14]. Since Benetazzo’s contributions, scholars have also begun utilizing 3D wave reconstruction to explore scientific questions within their respective fields. For instance, in a study of extreme sea waves, Benetazzo et al. examined the occurrence of rare giant waves within spatial and temporal samples [15]. Fedele et al. compared statistical data of sea waves across spatial and temporal samples [16]. In 2016, Benetazzo extended the WASS to mobile platforms and proposed a criterion for robust sea-surface estimation: without external ship-motion compensation, the field of view should include at least 16 two-dimensional waves [17]. Schwendeman and Thomson used a ship-borne stereo-vision system to examine the three-dimensional temporal evolution of breaking waves [18]. In 2017, Bergamasco optimized Benetazzo’s approach and released the first source-code version of WASS [19]. Guimarães provided the first stereo datasets collected across different sea areas and wave conditions, supplying valuable data for studies of sea-surface dynamics [20]. More recently, to accelerate stereo reconstruction, Bergamasco proposed WASSfast and later introduced deep learning-based improvements, while Sun achieved fast matching over large-area imagery using grid-based SiftGPU [21,22,23].

Stereoscopic vision wave measurement is increasingly being applied to mobile platforms. Vieira used smartphone cameras to retrieve sea-surface elevation information and later demonstrated the feasibility of a low-cost stereo-vision approach [24,25]. Greenberg explored a stereo-vision method for small unmanned surface vessels [26]. However, unlike stable shore- or tower-based settings, ship-borne observations are jointly influenced by platform motion, deployment constraints, sun glint, and foam-texture variability, causing stereo matching and 3D-reconstruction quality to fluctuate with observing conditions. Existing work has largely emphasized feasibility demonstrations, with limited, systematic quantification and engineering guidance on the deployment geometry and sampling parameters that govern measurement accuracy. Consequently, accuracy is hard to predict before deployment and difficult to reproduce across different mobile platforms.

To bridge these gaps, we propose a stereo-vision wave-observation scheme tailored to ship-borne mobile platforms and perform quantitative analyses of application-critical issues. By identifying appropriate deployment parameters, the scheme offers a feasible pathway for acquiring wave parameters on mobile platforms. The primary innovations and contributions are: (1) We develop a ship-borne stereo-vision wave-measurement apparatus and benchmark its performance against conventional wave-observation instruments. (2) We conduct sensitivity analyses of deployment parameters that govern accuracy, and we quantify their impacts on both measurement accuracy and the spatial pattern of errors. (3) We derive practical parameter-selection and deployment recommendations for ship-borne mobile platforms, providing quantitative guidance for future engineering deployment of stereo-vision wave measurements.

The subsequent sections are structured as follows: Section 2 describes the ship-borne experimental apparatus; Section 3 presents the experimental design and implementation; Section 4 analyzes the results; Section 5 discusses the findings; and Section 6 presents the overall conclusions.

## 2. Construction of Ship-Borne Stereoscopic Vision Experimental Apparatus

The ship-borne stereoscopic vision experimental apparatus is primarily composed of high-resolution cameras, complemented by a custom-designed exterior structure and a control program for synchronized acquisition, forming the hardware component of the experimental device. The software component consists of the WASS and a proprietary camera calibration program, which together enable the capture of wave images and the acquisition of spatiotemporal wave data.

### 2.1. Hardware Component

**Figure 1:**
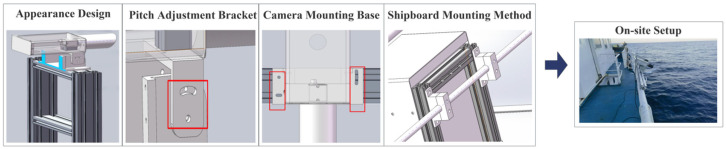
Schematic diagram of the hardware component of the ship-borne stereoscopic vision experimental apparatus.

The hardware component includes an image acquisition module, a waterproof protective casing, and mounting assemblies. Because ship motion can introduce image blur, the image acquisition module comprises a pair of Daheng industrial cameras (MER2-503-36U3M) with 8.3 mm fixed-focus lenses (Beijing Daheng Image Vision Co., Ltd., Beijing, China). Exposure and gain are set to fixed values to prevent auto-exposure-induced frame-to-frame brightness fluctuations. The cameras are connected to a computer via USB interfaces, enabling real-time acquisition of high-quality stereo wave image sequences. The waterproof protective casing is constructed from sheet metal and machined parts, with openings at the bottom for cable ingress and egress. The entire casing is mounted on an aluminum-alloy, pitch-adjustable bracket; the angle is set with screws and can be adjusted from 20° to 90°, providing a field of view that can cover a sea-surface area up to approximately 50 m from the cameras. The mounting assemblies consist of an extruded-profile support and heavy-duty clamps; the hardware is integrated on the profile support, whose height is adjustable to provide an optimal viewing angle. The profile support is secured to the deck railing using marine heavy-duty clamps to ensure system stability and safety. The hardware configuration of the experimental device is illustrated in Figure 1.

The apparatus control software is developed using the Daheng Galaxy SDK. Synchronized stereo image pairs are obtained via continuous streaming with triggered capture. Upon startup, the left and right cameras are opened in sequence by serial number and configured with a unified parameter set: continuous acquisition is enabled, trigger mode is activated, the pixel format is fixed to 8-bit grayscale, and exposure and gain are set to constant values to improve frame-to-frame consistency. During acquisition, each trigger produces one synchronized stereo pair. The acquired grayscale frames are demosaicked and converted to the target color space, and they are named and logged using device timestamps. To improve temporal alignment, we introduce a right-camera trigger delay (∆τ) within a software-triggering framework: the left camera is triggered first, and the right camera is triggered after a fixed interval ∆τ. Calibrating ∆τ compensates for the fixed trigger-to-exposure latency mismatch between the two cameras, making their exposure start times as consistent as possible. Following this procedure, the time offset between paired frames is kept within 3 ms.

### 2.2. Software Component

The software component of the experimental apparatus is developed based on the WASS [19]. This involves designing an intrinsic calibration program and perfecting the on-site calibration process to fine-tune the system, ensuring compatibility with the constructed hardware. Consequently, an integrated process from image acquisition to wave reconstruction can be achieved. The processing pipeline comprises four steps: (1) camera intrinsic calibration; (2) recovery of camera extrinsics; (3) 3D point-cloud reconstruction; and (4) sea-plane estimation. Steps (2)–(4) are performed using WASS.

The calibration of the camera’s internal parameters utilizes the Zhang Zhengyou calibration method [27]. This method involves capturing multiple images of a planar calibration board and using the feature points within these images to estimate the camera’s intrinsic parameters. This study presents an in situ calibration scheme. We developed an intrinsic-calibration tool using the OpenCV library, which builds the camera projection model by associating known 3D coordinates of the calibration target with their corresponding 2D image points. The intrinsics are then estimated via nonlinear optimization by minimizing the reprojection error between detected image points and those predicted by the projection model. This yields accurate intrinsic parameters (focal length, principal point, and distortion coefficients) and the corresponding intrinsic matrix for each camera. For calibration, each camera captures 60 images of a planar calibration target. The software flags frames with failed corner detection and automatically excludes them from intrinsic estimation, improving both accuracy and computational efficiency while maintaining an adequate number of valid samples.

The calibration of the camera’s external parameters relies on the WASS system. The core idea is to estimate the relative camera pose using a set of matched points between the two images. Feature points $p_{1}$......$p_{n}$ are extracted from the images captured by the left camera, and corresponding points ${p\prime }_{1}$.....${p\prime }_{n}$ are identified in the images from the right camera. The essential matrix E is estimated using the epipolar constraint (as shown in Equation (1)) [28], and subsequently, the rotation matrices R and translation matrices T are recovered through singular value decomposition [29]:

(1) $$p_{i}=K_{1}^{-T}EK_{2}^{-1}{p\prime }_{i}$$

The essential matrix, $E=\hat{T}R$, encodes the relative rotation (R) and the translation direction (T) between the two cameras. It represents the stereo relative pose in a compact 3 × 3 form and imposes the epipolar constraint: a point in one image must correspond to a point lying on the epipolar line in the other image. Here, $K_{1}$ and $K_{2}$ are the intrinsic matrices of the left and right cameras.

**Figure 2:**
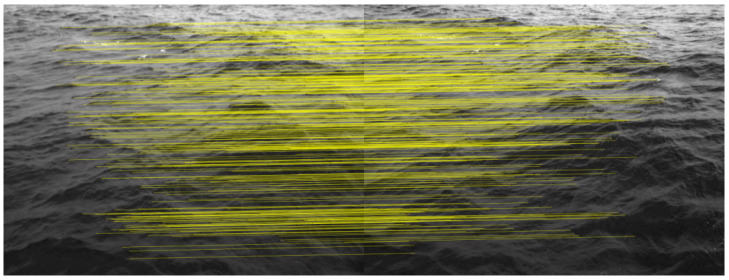
Feature matching in stereo images after RANSAC epipolar filtering.

To obtain correspondences from each stereo pair, WASS first extracts Speeded-Up Robust Features (SURF) following Bay et al. [30]. The image is partitioned into 16 blocks, and low-response feature points are iteratively pruned within each block, yielding 2600 retained features per image. For each feature, SURF estimates its dominant orientation and scale and computes a descriptor, which are then used for feature matching in later steps. For feature matching, WASS employs the correspondence-selection strategy of Albarelli et al., which is designed to identify reliable matches under motion [31]. In a game-theoretic formulation, candidate correspondences compete while reinforcing one another when they satisfy the same semi-local constraints, producing a compact set of mutually consistent matches. WASS then removes outliers using RANSAC-based epipolar filtering (Figure 2) and computes an initial essential matrix E for each stereo pair via Equation (1). Finally, sparse bundle adjustment (SBA) refines the RANSAC-filtered estimate of E, from which the camera extrinsic parameters are obtained [32].

**Figure 3:**
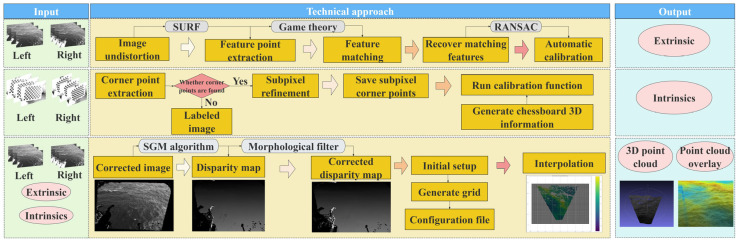
Technical roadmap for three-dimensional wave reconstruction.

The fundamental principle of three-dimensional wave reconstruction is based on the stereoscopic imaging model and the geometric relationships of its relative positioning, as illustrated in Figure 3. Once the pixel correspondence of a point across two cameras is established (i.e., the two projections of the same three-dimensional feature in the scene), the three-dimensional position of the point can be recovered by intersecting the rays emitted from both cameras. Specifically, each pair of stereo images is rectified using the external and internal calibration data of the stereo cameras. Subsequently, disparity maps are obtained using the Semi-Global Block Matching algorithm available in the OpenCV library [33]. This method corrects matching deviations around the whitecap areas and avoids introducing brightness equalization or complex pyramid search methods [34]. Because the disparity map tends to be unreliable near the boundaries of matched regions, we apply basic morphological filtering directly to the disparity map to suppress outliers. An initial 3D point cloud is then generated via triangulation. To further improve point-cloud quality, we remove depth outliers and discard points with very small intersection angles. In addition, exploiting the spatial continuity and smoothness of the sea surface, WASS applies an extra graph-based filtering step. A graph is built over the reconstructed 3D points, where vertices denote points and edges connect each point to its four nearest neighbors. Each edge is weighted by the absolute difference in the two endpoints’ coordinates along the camera z-axis. Edges in the highest 2% of this weight distribution are then pruned, as they indicate unusually large depth discontinuities and are treated as outliers. The associated points are subsequently removed, which improves the accuracy and robustness of stereo reconstruction.

Sea-plane estimation aims to transform the reconstructed 3D point cloud from the camera frame into a sea-surface-consistent reference frame. Because the camera is typically installed with a pitch angle and its axes are not strictly parallel to the mean horizontal sea surface, the mean sea plane is estimated from the point cloud. The 3D data are then rotated and translated so that the new vertical axis points upward, ensuring that subsequent sea-surface elevations are defined normal to the mean sea plane. After this step, the elevation values of the point cloud represent the actual sea-surface elevation. A detailed description of the coordinate-frame transformation procedure can be found in the paper by Benetazzo et al. [17].

### 2.3. Wave-Parameter Definitions and Computation Methods

As the most commonly used statistics in wave observations and quantities directly provided by most instruments, significant wave height ($H_{s}$) and significant wave period ($T_{s}$) are adopted as the primary evaluation metrics in this study (see Equations (2) and (3) for definitions):

(2) $$H_{s}=\frac{1}{N/3}\sum_{i=1}^{N/3}H_{i}$$

Here, N denotes the number of complete individual waves identified within the statistical time window, $H_{i}$ is the height of the i-th individual wave, and N/3 represents the number of waves included in the highest-one-third averaging.

(3) $$T_{s}=\frac{1}{N/3}\sum_{i=1}^{N/3}T_{i}$$

Here, N denotes the number of complete individual waves identified within the statistical time window, $T_{i}$ is the period of the i-th individual wave, and N/3 represents the number of waves included in the highest-one-third averaging.

At each sampling instant, 3D reconstruction yields a sea-surface point cloud over a given field of view, describing the instantaneous spatial form of the surface. To make the point-cloud output comparable with standard wave-observation metrics, we apply sea-plane estimation and extract an instantaneous elevation time series at the center of the field of view. This series is then used for wave analysis. With the open-source software Oceanlyz, wave height and wave period are calculated using the time-domain zero-crossing method, and significant wave height and significant wave period are derived according to Equations (2) and (3) [35].

To validate the accuracy of the above algorithm in estimating wave parameters, we use the Black Sea stereo-imaging dataset contributed by Guimarães [20]. Taking significant wave height as a representative metric, we analyze wave observations recorded by the stereo-vision system during 2011–2013 and quantify the error of Hs estimates from the stereo system relative to a wave gauge. The main parameters of the stereo-vision device and detailed field conditions are documented by Shokurov [36].

**Figure 4:**
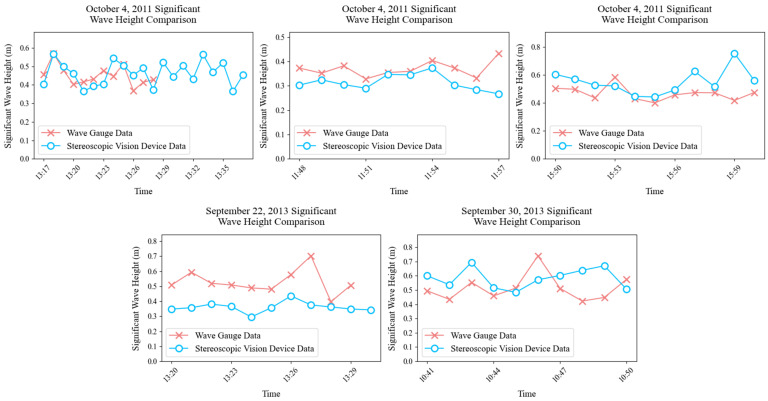
Comparison of significant wave heights over time.

As shown in Table 1, a comparison between the stereo camera system and the wave gauge indicates that their Hs estimates are similar in most cases, with some differences: the maximum deviation is 0.165 m, the minimum deviation is 0.015 m, the root mean square error (RMSE) is 0.1 m (computed relative to the wave gauge, indicating the error magnitude), and the mean error is 0.086 m (computed relative to the wave gauge by averaging the absolute error). Figure 4 compares the temporal evolution of significant wave height measured by the two instruments over the dates considered. Significant wave height was computed using a 10- or 20-min sliding window (depending on data availability) and updated every 1 min. By examining the significant wave height estimated by the two instruments, we find that the stereo camera system differs only slightly from the wave gauge, with the closest agreement observed at 13:00 on 4 October 2011. These results indicate that the proposed algorithm is feasible and yields accurate wave-parameter estimates, supporting ship-borne wave observations.

### 2.4. Experimental Apparatus Parameters Affecting Wave Observation Performance

In this section, we develop a device-parameter error model. Under a stereo geometry with epipolar rectification and calibrated intrinsic and extrinsic parameters, the depth Z is given by Equation (4).

(4) $$Z=\frac{f_{px}B}{d}$$

Here, Z denotes the depth of the target point along the principal optical axis in the camera coordinate system. This relationship holds, provided that the stereo system has completed intrinsic/extrinsic calibration and distortion correction and has been epipolar-rectified to satisfy standard stereo geometry. $f_{px}$ is the focal length in pixels, B is the baseline length (m), and d is the disparity (pixels). Under the triangulation model, d can be written as shown in Equation (5):

(5) $$d=2f_{px}\tan\left(\frac{\varphi }{2}\right)$$

In the equation, $f_{px}$ denotes the focal length in pixels (px), and φ denotes the convergence angle at the target point (i.e., the angle between the two camera rays at the target). Accordingly, under this geometric model, first-order error propagation of Z(d) yields the relative accuracy of the depth (Equation (4)):

(6) $$\frac{\sigma_{Z}}{Z}\approx \frac{\sigma_{d}}{d}=\frac{\sigma_{d}}{2f_{px}\tan\left(\frac{\varphi }{2}\right)}$$

The above error model applies under the assumptions that the stereo system has completed intrinsic calibration, extrinsic calibration, and distortion correction, and that the baseline length B and intrinsic parameters remain stable during acquisition. If pronounced image blur leads to matching failure, disparity uncertainty can increase substantially, and Equation (4), together with conclusions based on first-order error propagation, may no longer hold. As indicated by Equation (6), the accuracy of sea-surface elevation depends on the device’s deployment parameters. In ship-based applications, the principal parameters are the mounting height (H), the baseline length (B), and the camera look-down angle (θ). The deployment parameters are defined in the ship’s local horizontal coordinate system: the mounting height H is the vertical distance from the camera optical center to the mean sea surface; the baseline length B is the separation between the optical centers of the left and right cameras; and the camera look-down angle θ is the angle between the camera principal optical axis and the vertically downward direction. Here, φ, which affects the near–far extent of the imaged area, is influenced by B within the apparatus configuration and observation range considered in this study. However, in most shipborne deployments, the baseline length and mounting height usually cannot be adjusted independently; therefore, their combined metric B/H should be considered when assessing its effect on sea-surface elevation estimation. In addition, because wave parameters are characterized as statistical quantities, sampling frequency should also be included in the accuracy-assessment framework.

## 3. Experimental Design

To assess the wave-measurement capability of the experimental apparatus under different sea states, experiments were conducted at two sites: S1 (39°7.623′ N, 117°52.927′ E) and S2 (18°0.000′ N, 109°35.160′ E). S1 is located within Bohai Bay, where wind waves dominated during the campaign, and was used to test performance under relatively weak sea conditions. S2 is located in a more open sea area with relatively stronger waves and was used to evaluate system performance under sea states more representative of shipborne operation. Conventional wave-observation instruments were used as references, including the Haiyao wave buoy (South China Sea Institute of Oceanology, Chinese Academy of Sciences, Guangzhou, China), the OWB-BS wave buoy (National Ocean Technology Center, Tianjin, China), and an Acoustic Wave And Current Profiler (AWAC) (Nortek, Rud, Norway). The reference instruments deployed at each site are summarized in Table 2.

**Figure 5:**
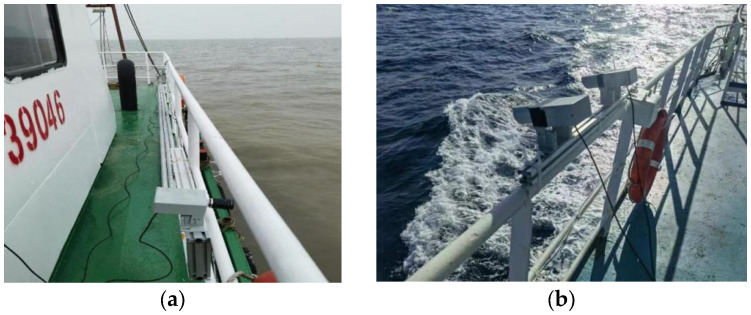
Schematic of the ship-borne stereoscopic vision experimental apparatus and its onboard deployment ((**a**): configuration at Site S1; (**b**): configuration at Site S2).

Throughout the experiments, we employed a fixed-point observation mode. Constrained by the test vessel’s physical conditions, the baseline length of the ship-borne stereo-vision apparatus was set to no more than 5 m, and the cameras were mounted approximately 6 m above the sea surface. The look-down angle was adjusted to 45° during the trials, providing sea-surface coverage out to approximately 60 m. The deployment configuration is shown in Figure 5.

(1) Experiment I: Assessing the apparatus’s measurement performance across different time windows.

At site S1, we conducted an intercomparison of multiple wave-measurement instruments over a single time window. Concurrent measurements were collected with an AWAC, an OWB-BS wave-spectrum buoy, and a Haiyao wave buoy, and the accuracy of the instruments was cross-validated. At site S2, using the Haiyao wave buoy as the reference, we performed both a single-window intercomparison and an extended intercomparison over continuous periods.

(2) Experiment II: Assessing the impact of the baseline-to-height ratio on measurement accuracy.

The experiment was conducted at site S2. With the mounting height held constant, the baseline-to-height ratio was varied by adjusting the baseline length, which ranged from 0.5 to 3.0 m. During the baseline adjustments, concurrent measurements were collected with the Haiyao wave buoy.

(3) Experiment III: Assessing the impact of sampling frequency on measurement accuracy.

At S1, the stereoscopic vision experimental apparatus acquired data at sampling frequencies of 1 Hz and 20 Hz (in separate runs), with concurrent measurements from an AWAC, an OWB-BS wave-spectrum buoy, and a Haiyao wave buoy. At S2, the apparatus operated at 10 Hz, and concurrent measurements were collected with the Haiyao wave buoy.

(4) Experiment IV: Assessing the spatial distribution of measurement accuracy across the observation area.

Using continuous-observation data from site S2, each point-cloud frame was gridded onto a two-dimensional plane at 0.5 m resolution. For each grid cell, the mean elevation of points within the cell was taken as the instantaneous wave height. On this basis, we estimated the significant wave height for each cell along the time series, with the Haiyao wave-buoy observations used as a reference, and we analyzed the spatial distribution of measurement accuracy across the observation area.

## 4. Results and Analysis

### 4.1. Accuracy Assessment of the Ship-Borne Stereoscopic Vision Apparatus

The intercomparison results of Experiment I are summarized in Table 3. The significant wave height deviation and significant wave period deviation are defined as the absolute differences between the experimental apparatus estimates and the corresponding values from each reference instrument. At site S1, the measurements from the three reference instruments were highly consistent: the range of significant wave height across instruments did not exceed 0.03 m (the difference between the maximum and minimum significant wave heights measured by the three reference instruments), and the sample standard deviation did not exceed 0.015 m. This multi-instrument consistency provides a sound basis for subsequent bias assessment of the experimental apparatus. For the stereo-vision device (20 Hz) at 17:19, the results were accurate: the maximum deviation in significant wave height was 0.14 m, and the maximum deviation in significant wave period was 0.54 s. These findings confirm that the constructed stereo-vision experimental apparatus achieves high measurement accuracy.

**Figure 6:**
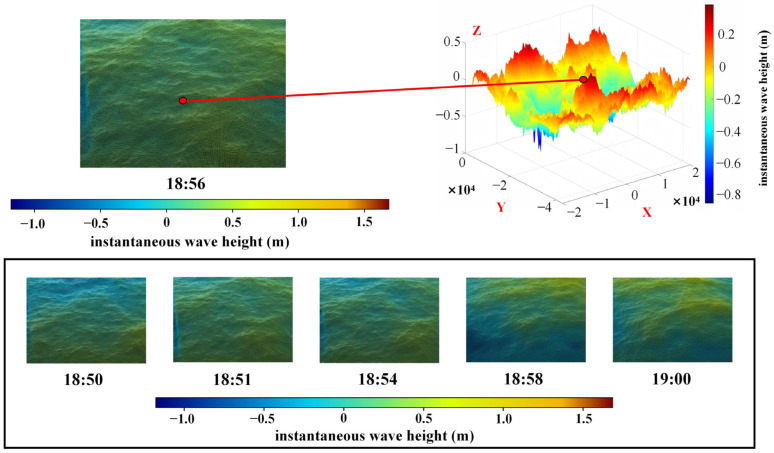
Reconstruction effect of sea waves by the stereoscopic vision experimental apparatus.

**Figure 7:**
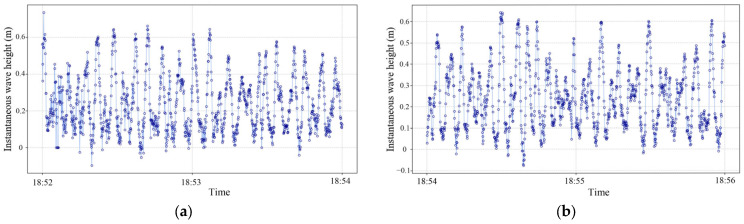
Reconstruction effect of sea waves by the stereoscopic vision experimental apparatus. (**a**) Instantaneous wave height map for the time period from 18:52 to 18:54; (**b**) Instantaneous wave height map for the time period from 18:54 to 18:56.

At site S2, the stereoscopic vision device collected multiple datasets at different times for targeted analysis. Figure 6 illustrates the reconstructed waves using the 18:50–19:00 dataset and extracts the instantaneous wave height at the center point. The time series of the center-point instantaneous wave height is shown in Figure 7. Based on these data, the stereo-vision experimental apparatus computed a significant wave height of 1.12 m at 19:00, which differs by 0.03 m from the Haiyao wave buoy measurement at the same time.

**Figure 8:**
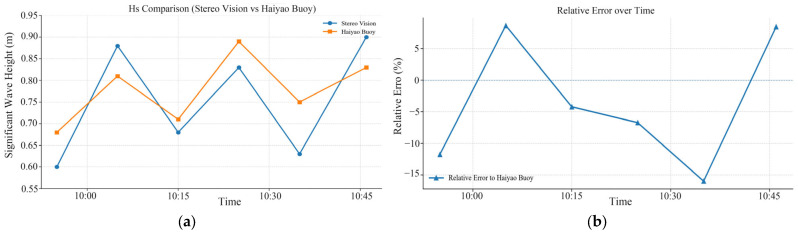
Observational results: (**a**) comparison of significant wave height over the time period; (**b**) temporal evolution of the relative error over the same period.

Figure 8a compares the temporal variation of significant wave height measured by the two instruments over the period of 09:45–10:45. During this period, the two time series exhibit consistent trends, with instantaneous deviations ranging from 0.03 m to 0.12 m. Figure 8b shows the evolution of the relative error: values are generally within ±10%, with the largest negative deviation at 10:35 and the largest positive deviation at 10:05. Using the Haiyao wave buoy as the reference, the stereo-vision experimental apparatus has a mean bias of −0.025 m (the arithmetic mean error relative to the Haiyao wave buoy) and a root mean square error (RMSE) of 0.076 m (computed with the Haiyao wave buoy as the reference, reflecting the error magnitude).

The discrepancies likely stem from differences in measurement principles and implementation. The stereoscopic vision device estimates wave height and period from image-derived surface features, making it more sensitive to ambient lighting, camera angle, and field of view. In contrast, the Haiyao wave buoy directly senses vertical sea-surface motion with physical sensors, yielding more stable measurements that are less affected by external conditions. Moreover, surface reflections (glare) or strong, irregular seas can hinder feature detection and reduce the accuracy of the stereoscopic vision device.

### 4.2. Impact of Parameter Settings on Measurement Accuracy

Equation (6) confirms a correlation between the device’s parameter settings and measurement accuracy. Accordingly, we first examined the baseline-to-height ratios used in prior studies, as summarized in Table 4.

The observations from Experiment II (Table 5) further validate the baseline-to-height ratio relationship.

Based on the computed relative errors, the dependence on the baseline-to-height ratio is non-monotonic, revealing a clear optimal range. Notably, this optimal interval is empirical and may change with the optical system characteristics and sea-state conditions. The relative error in significant wave height is minimal at B/H = 0.16 and 0.25. When B/H falls below this range (e.g., B/H = 0.10), the shortened baseline reduces measurable disparity and increases the error. At B/H = 0.41, the relative error grows substantially, and the estimation fails at B/H = 0.50.

Table 6 summarizes feature-match counts for eight images in Experiment II across baseline lengths. High match counts are observed at 0.5 m and 1.0 m; counts drop markedly at 2.0 m and 2.5 m; and at 3.0 m, matches are very sparse. Taken together, Table 5 and Table 6 indicate that when B/H is 0.16–0.25, the match count remains sufficient, and the significant wave height error is relatively small. When the baseline increases further, the number of feature matches decreases sharply, which degrades 3D reconstruction quality and leads to a pronounced increase in significant wave height error. This confirms that B/H admits an optimal operating range; beyond this range, the apparatus accuracy drops significantly.

Because wave parameters are computed as statistical quantities, sampling frequency also affects measurement accuracy. Based on Experiment III (Table 3 and Table 7), the ship-borne stereo-vision apparatus at S1 produced accurate observations at 20 Hz. In contrast, at 1 Hz, the estimated wave parameters exhibited noticeably larger errors relative to 20 Hz. At S2, with a sampling frequency of 10 Hz, the deviations in significant wave height across different time windows remained within 0.12 m.

**Figure 9:**
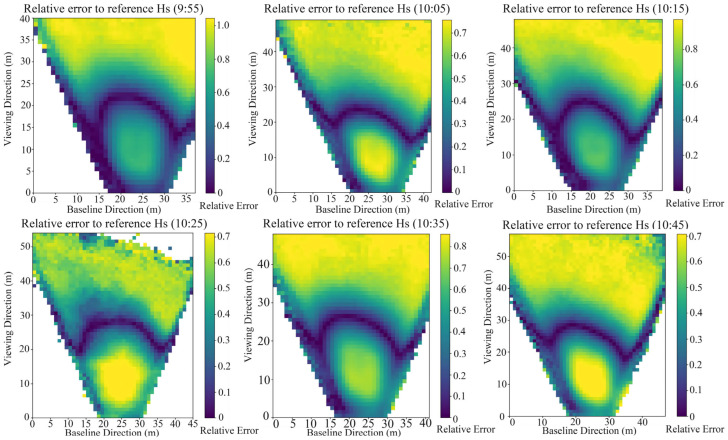
Direction-dependent spatial distribution of the relative error of significant wave height across the observation area.

Experiment IV maps the spatial distribution of measurement accuracy across the observation area (Figure 9). Here, the baseline direction is defined as the line connecting the optical centers of the left and right cameras. The direction orthogonal to the baseline within the observation plane is defined as the baseline-normal direction. With B/H = 0.1, the relative error of significant wave height is direction dependent: along the camera baseline, the error varies smoothly; along the baseline normal, it generally decreases from the near side (camera side) toward the far side and then increases. A baseline-parallel low-error band is identifiable, characterized by low error levels and slow spatial variation; outside this band, errors rise markedly. This behavior arises because, assuming an approximately planar sea surface and a fixed baseline, the convergence angle φ changes slowly along the baseline direction, but along the baseline normal, it decreases as the observation point moves from the near side to the far side.

As indicated by Equation (6), if $\sigma_{d}$ is approximately constant, the relative depth accuracy decreases monotonically with decreasing φ. Consequently, error gradients are small along the baseline, whereas across the baseline, the error tends to increase from the near side to the far side. In practice, however, $\sigma_{d}$ is not constant; it depends on the stability of the matching algorithm, scene texture and noise, motion blur, and extreme viewing angles. Focusing on deployment factors, the near side is affected by extreme obliquity and perspective foreshortening, which enhance texture anisotropy; together with vessel occlusion and local flow disturbances, these effects flatten the matching-cost curve and raise $\sigma_{d}$. On the far side, $\sigma_{d}$ may be smaller, but geometric effects amplify the relative error as φ→0. In our experimental apparatus, these two effects increase errors at the near and far limits, respectively. Consequently, the moderate-angle region preserves sufficient geometric information while being less affected by occlusion and oblique viewing, yielding a low-error band.

**Figure 10:**
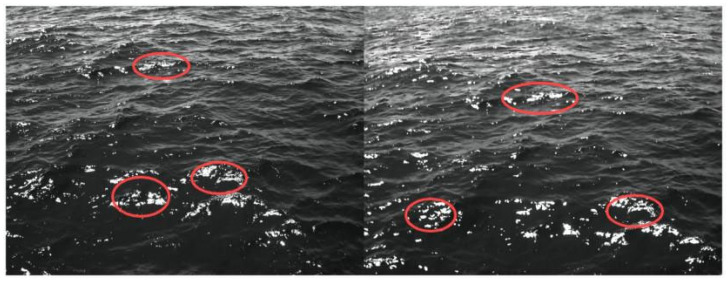
Waves with specular reflection.

Additionally, when setting the system’s pitch (look-down) angle, avoid imaging directly toward the sun. In the S2 trials, sun-facing acquisitions produced significant 3D-reconstruction errors: sunlight-reflecting areas of the wave field appeared as obvious gaps after reconstruction. The underlying cause is that wave-induced surface undulations create intense specular highlights at varying angles and positions. These highlights can exceed the camera sensor’s dynamic range, leading to overexposure and texture loss and appearing as saturated bright patches (Figure 10). Lacking recoverable detail, such regions cannot be reliably reconstructed in 3D, thereby degrading the accuracy of the results.

## 5. Discussion

In shipborne settings, stereo vision is often limited by platform motion and varying imaging conditions, including sea-surface glare, splash occlusion, and texture changes, which can undermine stereo-matching stability and the consistency of 3D reconstruction. The apparatus and results reported here depend on the sea states and observation conditions encountered during the trials. Accordingly, we discuss the findings based on observations from the proposed shipborne stereo-vision apparatus at sites S1 and S2.

While WASS operates normally across sampling rates, overly low sampling frequencies markedly degrade the accuracy of wave-parameter estimates produced by the experimental apparatus. For a stereoscopic vision device, low frame rates typically require longer exposures, so sea-surface and platform motion induce blur and increase feature-matching noise. When significant wave height is computed over short windows (e.g., 10 min), such instantaneous errors are more readily amplified. Hence, very low sampling frequencies are unsuitable for stable estimation of wave parameters.

Deployment parameters are a key determinant of device accuracy. The baseline-to-height ratio has a viable range: within this range, significant wave height errors remain stable; beyond it, errors rise sharply and reconstruction failures may occur. The underlying mechanism is that as the baseline grows past the viable B/H range, image feature-match counts drop markedly, which degrades stereo reconstruction and, in turn, the accuracy of the derived wave parameters. The pitch angle defines the observation footprint. If the angle is too large, images contain extraneous scene content and the algorithmic workload increases; if it is too small, as indicated by the Section 4.2 maps of the relative error of significant wave height, wave imagery is prone to vessel occlusion and flow disturbances. Moreover, sun-induced specular reflections from the sea surface further degrade the accuracy of the experimental apparatus.

Overall, sampling frequency, deployment parameters, and lighting conditions affect the accuracy of wave reconstruction by the experimental apparatus, with the effects being more pronounced under mild (low-energy) sea states. Section 4.2 confirms a low-error band within the imaged area, which identifies the position of optimal accuracy for instantaneous elevation estimates. To expand this band, we recommend the following deployment settings, grounded in our data and geometric analysis: a sampling frequency of 10 Hz; a baseline-to-height ratio of 0.1–0.3; and a look-down (pitch) angle of 45–60°. It should be noted that the above parameter ranges are empirical recommendations based on the optical system, installation setup, and sea conditions in this study. While they are broadly applicable to WASS deployment on mobile platforms, differences in the camera, lens parameters, and platform characteristics may shift the low-error band observed here. Therefore, engineering deployments should verify and adjust these settings according to the specific platform and on-site conditions.

## 6. Conclusions

This study integrates the Wave Acquisition Stereo System (WASS) into a stereo-vision wave-measurement scheme, develops a ship-borne experimental apparatus, and analyzes the key techniques required for ship-borne wave measurements. Sea trials yielded reliable wave-observation results. Compared with prior studies, this work primarily contributes a ship-borne stereo-vision wave-measurement scheme and validates it against conventional wave-observation instruments under real shipborne trial conditions. In addition, we perform sensitivity analyses of key deployment parameters for mobile platforms, provide practical parameter recommendations, and describe the spatial characteristics of measurement errors. The main conclusions are as follows:

Using a pair of industrial cameras with 8.3 mm fixed-focus lenses integrated with the Wave Acquisition Stereo System (WASS), we developed a ship-borne experimental apparatus that synchronizes wave-image acquisition and reconstructs the instantaneous sea surface in 3D, enabling real-time measurement of instantaneous wave height. Validation at two sea sites shows that, relative to conventional wave buoys, errors in significant wave height do not exceed 0.14 m and are generally within ±10%, demonstrating the feasibility of ship-borne stereo-vision wave measurement.

Low image sampling frequencies (e.g., 1 Hz) increase motion blur and feature-matching noise, which are amplified when statistics are computed over short windows. In contrast, higher sampling frequencies (e.g., 10 Hz) better capture the evolution of the sea surface and improve the accuracy and stability of wave-parameter estimates.

The baseline-to-height ratio (B/H) is nonlinearly related to the relative error of significant wave height. For B/H = 0.10–0.33, relative errors are modest and similar, whereas B/H > 0.33 degrades feature matching. A moderate look-down angle maintains effective coverage and sufficient texture; in deployment, avoid strong specular reflections, occlusions, and far-field clutter. Overall, measurement performance is jointly governed by sampling frequency and geometric deployment settings.

The wave-observation experiments in this study were conducted in a fixed-point mode, and motion effects during vessel transit were only partially considered. Under sailing conditions, six-degree-of-freedom vessel motions may further reduce stereo-matching stability and degrade 3D reconstruction quality. Future work will target transit operation and more complex sea states by introducing image-stabilization and motion-compensation algorithms. Extensive sea trials will be conducted to systematically quantify motion impacts on measurement performance and to further extend wave-spectrum extraction methods.

## Figures and Tables

**Table 1:** Significant wave heights in the same sea area measured by stereo cameras and wave gauge.

Recording Date	Duration (min)	Effective Wave Height by Camera (m)	Effective Wave Height by Wave Gauge (m)	Deviation (m)
4 October 2011	30	0.445	0.460	0.015
4 October 2011	19	0.312	0.371	0.059
4 October 2011	30	0.587	0.492	0.095
22 September 2013	30	0.357	0.522	0.165
30 September 2013	30	0.598	0.502	0.096

**Table 2:** Wave-observation instruments by sea area.

Observation Sea Area	Equipment	Observation Principle	Observation Range	Resolution
S1	Haiyao Wave Buoy	Inertial wave sensing	Significant wave height: 0.2–20 m; period: 2.0–20 s; direction: 0–360°.	Significant wave height: ±(0.03H) m; period: ±0.1 s; direction: ±1°.
	OWB-BS Wave-Spectrum Buoy	Inertial wave sensing	Significant wave height: 0.2–20 m; period: 2.0–20 s; direction: 0–360°.	Significant wave height: ±(0.1 + 0.005H) m; period: ±0.5 s; direction: ±0.5°.
	Acoustic Wave and Current Profiler	Pressure and AST (Acoustic Surface Tracking)	Wave height: ±15 m; period: 1–50 s.	Directional resolution: 0.1°; pressure sensor: 0.5% full scale.
S2	Haiyao Wave Buoy	Inertial wave sensing	Significant wave height: 0.2–20 m; period: 2.0–20 s; direction: 0–360°.	Significant wave height: ±(0.03H) m; period: ±0.1 s; direction: ±1°.

**Table 3:** Observational results of wave parameters at site S1.

Equipment	Significant Wave Height (m)	Deviation in Significant Wave Height (m)	Significant Wave Period (s)	Deviation in Significant Wave Period (s)
Stereoscopic vision device (20 Hz)	0.38		1.96	
OWB-BS wave-spectrum buoy	0.25	0.13	2.5	0.54
Haiyao wave buoy	0.24	0.14	2.3	0.34
AWAC	0.27	0.11	1.69	0.2

**Table 4:** Deployment parameters of the stereo-vision apparatus reported in previous studies.

Observation Site	Record Time	Baseline (m)	Height (m)	Height/Baseline
Venice	September 2004			0.1
Black Sea	1 October 2011	2.03	11	0.18
	4 October 2011	2.03	11	0.18
	22 September 2013	1.87	11	0.17
	25 September 2013	1.87	11	0.17
Adriatic Sea	27 March 2014	2.5	12.5	0.2
	5 March 2015	2.5	12.5	0.2
	15 May 2014	2.5	12.5	0.2
Yellow Sea	13 May 2017	5.04	33	0.15
Caspian Sea	3 January 2018	5.4	45	0.12

**Table 5:** Observational results for different baseline lengths in Experiment II.

Record Time	Baseline (m)	Baseline/Height	Relative Error of Significant Wave Height
13 June 2024 9:55	0.6	0.1	11.8%
13 June 2024 14:18	1	0.16	6.7%
13 June 2024 15:49	1.5	0.25	6.8%
13 June 2024 17:18	2	0.33	13.6%
13 June 2024 18:00	2.5	0.41	48.5%
13 June 2024 18:27	33	0.5	Erro

**Table 6:** Number of feature point matches for eight images at six different baseline lengths.

Baseline (m)	Image1	Image2	Image3	Image4	Image5	Image6	Image7	Image8
0.6	398	384	397	397	421	395	421	417
1.0	321	311	351	292	310	284	274	308
1.5	211	266	217	217	228	215	188	242
2.0	110	166	118	136	126	140	134	166
2.5	109	123	130	111	92	129	105	118
3.0	30	23	8	17	14	7	8	15

**Table 7:** Intercomparison results for the stereo-vision experimental apparatus (1 Hz) at site S1.

Equipment	Significant Wave Height (m)	Deviation in Significant Wave Height (m)	Significant Wave Period (s)	Deviation in Significant Wave Period (s)
Stereoscopic vision device (1 Hz)	0.66		5.4	
OWB-BS wave-spectrum buoy	0.2	0.46	2.6	2.8
Haiyao wave buoy	0.19	0.47	2.4	3
AWAC	0.19	0.47	1.96	3.44

## Data Availability

The data presented in this study are available upon reasonable request from the corresponding author. The data are not publicly available due to confidentiality restrictions related to ship operation routes and institutional regulations.
